# Factors hindering health care delivery in nomadic communities: a cross-sectional study in Timbuktu, Mali

**DOI:** 10.1186/s12889-021-10481-w

**Published:** 2021-02-28

**Authors:** Moussa Sangare, Yaya Ibrahim Coulibaly, Siaka Yamoussa Coulibaly, Housseini Dolo, Abdoul Fatao Diabate, Kueshivi Midodji Atsou, Abdoul Ag Souleymane, Youssouf Ag Rissa, Dada Wallet Moussa, Fadimata Wallet Abdallah, Massitan Dembele, Mahamadou Traore, Tieman Diarra, William R. Brieger, Sekou Fantamady Traore, Seydou Doumbia, Samba Diop

**Affiliations:** 1 Mali International Center for Excellence in Research (ICER), University of Sciences, Techniques, and Technologies of Bamako (USTTB), Bamako, Mali; 2 Interdisciplinary School of Health Sciences | Faculty of Health Sciences, University of Ottawa, 75, av. Laurier Est, Ottawa ON K1N 6N5, Canada; 3 General Directorate of Health and Public Hygiene, Ministry of Health and Social Affairs of Mali, Bamako, Mali; 4 World Health Organization (WHO), Regional Office for Africa, Cite du Djoue, PO Box 06, Brazzaville, Congo; 5 Department of International Health, Health System Program, The Johns Hopkins Bloomberg School of Public Health, Baltimore, MD 21205 USA

**Keywords:** Nomadic, Access, Healthcare, Delivery, Timbuktu, Mali

## Abstract

**Background:**

In Mali, nomadic populations are spread over one third of the territory. Their lifestyle, characterized by constant mobility, excludes them from, or at best places them at the edge of, health delivery services. This study aimed to describe nomadic populations’ characteristics, determine their perception on the current health services, and identify issues associated with community-based health interventions.

**Methods:**

To develop a better health policy and strategic approaches adapted to nomadic populations, we conducted a cross-sectional study in the region of Timbuktu to describe the difficulties in accessing health services. The study consisted in administering questionnaires to community members in the communes of Ber and Gossi, in the Timbuktu region, to understand their perceptions of health services delivery in their settings.

**Results:**

We interviewed 520 individuals, all members of the nomadic communities of the two study communes. Their median age was 38 years old with extremes ranging from 18 to 86 years old. Their main activities were livestock breeding (27%), housekeeping (26.4%), local trading (11%), farming (6%) and artisans (5.5%). The average distance to the local health center was 40.94 km and 23.19 km respectively in Gossi and Ber. In terms of barriers to access to health care, participants complained mainly about the transportation options (79.4%), the quality of provided services (39.2%) and the high cost of available health services (35.7%). Additionally, more than a quarter of our participants stated that they would not allow themselves to be examined by a health care worker of the opposite gender.

**Conclusion:**

This study shows that nomadic populations do not have access to community-based health interventions. A number of factors were revealed to be important barriers per these communities’ perception including the quality of services, poverty, lifestyle, gender and current health policy strategies in the region. To be successful, future interventions should take these factors into account by adapting policies and methods.

**Supplementary Information:**

The online version contains supplementary material available at 10.1186/s12889-021-10481-w.

## Background

In rural areas of Mali, especially in the northern part of the country, local populations experience the highest infant mortality rates (191 per 1000 births). In other words, one out of five Malian children in these areas dies before reaching 5 years of age [1]. The distribution of health facilities throughout the country is disparate and based not only on demographics but also on politics and socio-economic aspects [2]. Indeed, health services and human resources are generally concentrated in the populated urban areas of the country. As an illustration, 57% of the medical doctors, 47% of the nurses and 64% of the midwives are currently working in the capital city, Bamako [3].

On the fringe of the Sahara desert in the Sahel region of West Africa, 65% of Mali’s land is desert or semi-desert [4]. Wealth and natural resources are unequally distributed across the country. Those living in the northern regions of the country suffer from recurring periods of drought and widespread food shortages [5, 6].

An estimated 50–100 million people or 30–60% of the world’s nomadic and semi-nomadic people live in Africa. Nomadism is common in many Sub-Saharan African countries including Mali [7–9]. Nomadic populations have less access to health services compared to the general population [10]. They are also disproportionately vulnerable to infectious diseases such as malaria, tuberculosis, Guinea worm disease, leishmaniasis, onchocerciasis, schistosomiasis, soil-transmitted helminthiases, brucellosis and trachoma [10].

Little is known on how to efficiently provide essential healthcare services to nomadic populations and those living in remote rural areas [11]. The constant mobility of nomadic populations excludes them, or at best places them at the edge of health delivery services. Nomadic community health workers (CHWs) recruitment, training and their support by the community constitute an additional challenge [12]. Preliminary data from this study showed that cultural and socio-economic factors such as gender, lifestyle, proximity and constant mobility with animals affect access to health interventions [13]. This study aimed to describe nomadic populations’ characteristics and determine factors limiting nomads’ access to current health services and identify accessibility-related health intervention issues among the nomads.

## Methods

### Study setting

The study was conducted in the Timbuktu region, in the commune of Ber, in the health district of Timbuktu and the commune of Gossi in the health district of Gourma Rharous, both of which are located at about 900 km from Bamako, the capital city of Mali [4].

### Study population

The study was performed among *Kel Tamasheq*, Songhai, Arab, Fulani and Bozo people. The main lifestyle of these populations is pastoralism that requires a nomadic lifestyle for a significant part of the community. Nomadic populations settle in small groups of 25–35 people in a campsite for a relatively long period of time (20–30 days) and then move from one seasonal grazing area to another without specific directions. Movement is driven by the grazing. Each campsite is composed of around 3–5 families with 5 to 7 households each living in 4–6 tents around a leader who is usually the eldest family member. They settle with their animals around an oasis in transhumance between the northern and southern parts of the country depending on the season and availability of pasture for their animals. With these conditions and cultural values, nomads prefer not to stay in these communities due to the constant search for new pastures for livestock. They spend most of their time and effort caring for their animals. Therefore, it seems as if the welfare and the health of their animals is as important as their own wellbeing [14].

### Study design

We conducted a cross-sectional study from January to March 2011 in the communes of Ber and Gossi by administrating a questionnaire to community members. In each commune, we went to the different villages and nomadic camps to interview people. When we arrived in a camp we explained the study and among the volunteers, 3 or 4 people were randomly selected. In the villages, from the list of families, 4 or 5 families were selected and 3 or 4 people from these families were selected as volunteers. It should be noted that in the study sites the size of the villages did not exceed 15–20 families. Only volunteers of 18 years of age and above who were able to give informed consent were included in the study.

### Sample size and statistical considerations

The sample size has been estimated by using Epi Info software; assuming 95% confidence level, power of 80 and 20% of no access in healthcare among the nomadic population. The level of significance was set at 0.05 (two-tailed). Data were analyzed using the statistical package for social sciences (SPSS) version 20.0 (https://www.ibm.com/analytics/spss-statistics-software) and chi-square tests performed to compare proportion as appropriate. The graphs were generated by using GraphPad Prism 8 (https://www.graphpad.com). Missing data were not included in the analysis. The missing data that were not considered in the analysis because they were very few. Only four (4) subjects who did not complete the questionnaire so we decided not to consider the incomplete information of these subjects in the analysis.

### Data collection methods and instruments

A questionnaire was administrated to community members by well-trained investigators. The content of the questionnaire was related to health problems specific to the context of nomadic communities, such as the lack of medicines or the inadequacy of modern means of transport such as vehicles and ambulances. For example, pregnant women with complications during maternal labor and delivery are transported in carts pulled by donkeys.

### Questionnaire development

For the questionnaire development (Additional file 1), we mainly focused on the study objectives to generate questions that could provide with accurate and complete information specific to the nomadic context of northern Mali. After the questionnaire development, it was reviewed by the senior researchers and then tested in Bamako during the simulation sessions. The shortcomings were considered before the actual field survey phase. The questionnaire development was done to get insight about nomads’ access to and use of available health care based on health belief model theory [15]. It was entirely made up by the study investigators for the purpose of this study.

## Results

We included a total of 520 participants in this study. The sex ratio of participants was 1.34 in favor of men with a median age of 38 [18–86] years. The 31–45 years group was the most represented among the study population. Most of the participants were livestock breeders (22.88%), housekeepers (20.77%) and traders (8.46%). Among them, livestock breeding, and housekeeping were the most frequent respectively for men and women (Table 1). Other occupations have been cited including craftsmanship, agriculture, civil servant and marabouts (8.27%).

**Table 1:** Characteristics of the study population

Characteristics	Ber	Gossi	Overall
	n (%)	n (%)	N (%)
**Male/Female (Ratio)**	164/95 (1.72)	134/127 (1.05)	298/222 (1.34)
**Median age (min; max)**	38 (18–86)	38 (18–86)	38 (18–86)
**Age-groups (in years)**			
18–30	107 (41.31)	75 (28.74)	182 (35)
31–45	89 (34.36)	101 (38.7)	190 (36.54)
≥ 46	63 (24.32)	85 (32.57)	148 (28.46)
Total	259 (100)	261 (100)	520 (100)
**Marital status**			
Single	41 (15.83)	20 (7.66)	61 (11.73)
Divorced	10 (3.86)	13 (4.98)	23 (4.42)
Married	159 (61.39)	181 (69.35)	340 (65.38)
Separated	9 (3.47)	7 (2.68)	16 (3.08)
Widowed	5 (1.93)	13 (4.980	18 (3.46)
Unknown	35 (13.51)	27 (10.34)	62 (11.92)
Total	259 (100)	261 (100)	520 (100)
**Education**			
*Kel Tamasheq* alphabet	0 (0)	4 (1.53)	4 (0.77)
College	2 (0.77)	0 (0)	2 (0.38)
Arabia	51 (19.69)	48 (18.39)	99 (19.04)
Primary school	37 (14.29)	20 (7.66)	57 (10.96)
Illiterate	145 (55.98)	187 (71.65)	332 (63.85)
Secondary	18 (6.95)	2 (0.77)	20 (93.85)
University	6 (2.32)	0 (0)	6 (1.15)
Total	259 (100)	261 (100)	520 (100)
**Main occupation**			
Livestock breeding	50 (19.31)	69 (26.44)	119 (22.88)
Trading	35 (13.51)	9 (3.45)	44 (8.46)
Housekeepers	41 (15.83)	67 (25.67)	108 (20.77)
Others	36 (13.90)	7 (2.68)	43 (8.27)
Total	259 (100)	261 (100)	520 (100)

Others = craftsmanship, agriculture, civil servant, marabout; min = minimum; max = maximum

In both Ber and Gossi, 63% of those surveyed in this study were illiterate. The other types of education among the participants in both cities were literacy in the Arabic language or primary school level (respectively 19.04 and 10.96% of those surveyed in this study). In our study, most of the participants (65.38%) were married, followed by single (11.73%) and divorced (4.42%). Approximately 11.92% of the participants did not wish to give their marital status (Table 1).

The distance to the healthcare facility was not excessive for 40% of our participants. With respect to cost, there was a huge difference between those who thought that health care costs were not too high (48.08%), and those who thought it was too expensive (27.50%). Most of participants (45.38%) said that the quality of health care was not good while 30.19% thought it was good (Table 2).

**Table 2:** Perception of the study population about some factors that decrease the medical centers frequentation (distance, cost, healthcare quality)

Most factors cited	n	(%)
**Distance (very far)**		
No	208	40.00
Yes	185	35.58
No respondents	127	24.42
**Total**	**520**	**100.00**
**Cost (very high)**		
No	250	48.08
Yes	143	27.50
No respondents	127	24.42
**Total**	**520**	**100.00**
**Healthcare quality (good)**		
No	236	45.38
Yes	157	30.19
No respondents	127	24.42
**Total**	**520**	**100.00**
**Lack of funding**		
No	76	14.62
Yes	317	60.96
No respondents	127	24.42
**Total**	**520**	**100.00**

Lack of resources (financial and mean of transportation) was an obstacle for 60.96% of participants. This rate was statistically significant compared to the rest of the participants (14.62%) who said it was not. More than 63% of the study population had a monthly income less than $120 US Dollars (Table 3).

**Table 3:** Average monthly income of respondents by gender

Average monthly income in USD	Female		Male	
	n	%	n	%
Unknown	76	34.23	46	15.44
< 120	137	61.71	192	64.43
120–200	6	2.70	46	15.44
201–350	2	0.90	10	3.36
351–500	1	0.45	3	1.01
> 500	0	0	1	0.34
**Total**	**222**	**42.69**	**298**	**57.30**

*USD* United States Dollars

**Fig. 1:**
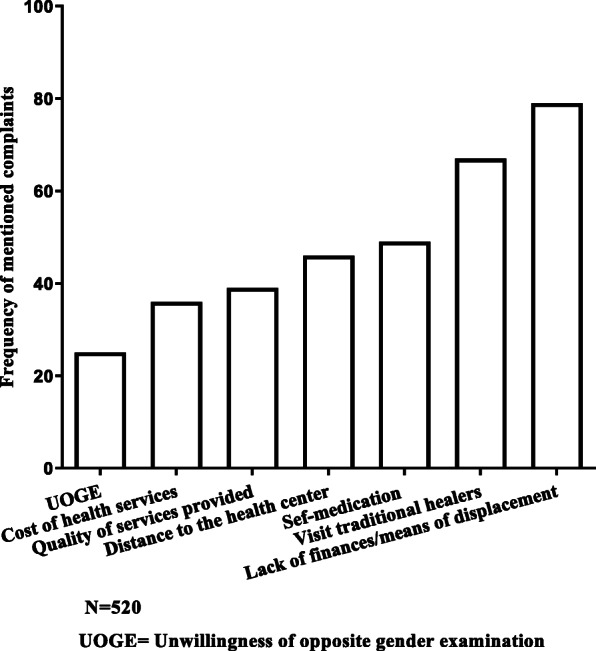
Frequency of the most common barriers to nomads’ access to health services mentioned by the respondents

**Fig. 2:**
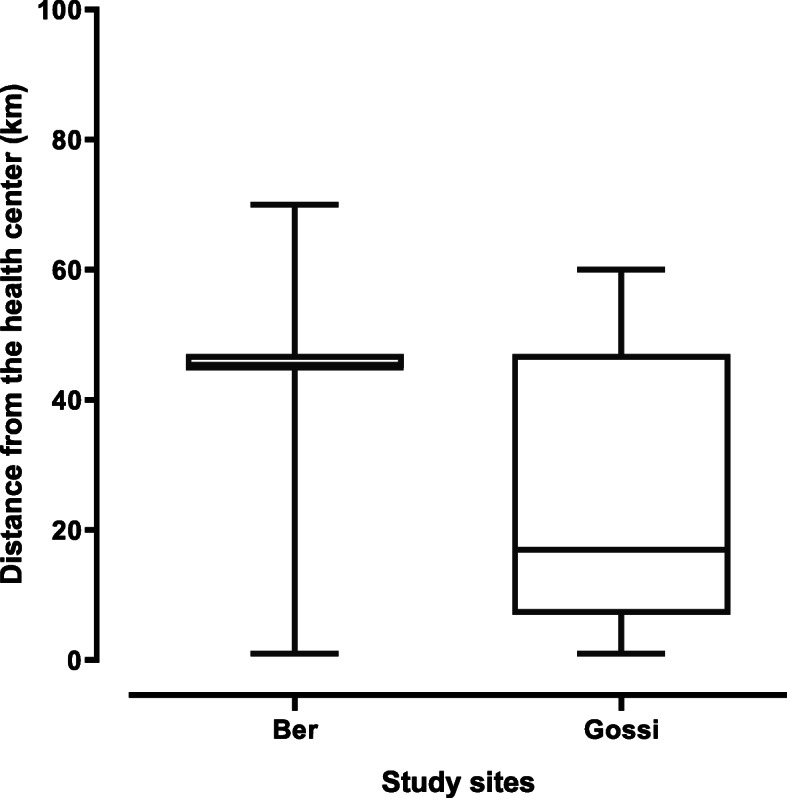
Variation of the median distance between respondents’ location and the closest health center per study site

The most common barriers to nomads’ access to health services included healthcare cost, means of transportation, visits to traditional healers, unwillingness of opposite gender medical examination and distance to the health center. Healthcare cost, means of transportation, and visit to traditional healers were the most frequent barriers reported (Fig. 1). Participants emphasized the lack of financial resources (60.96%). Some of our participants lived in campsites located at an average distance of 40 km in Ber and 23.19 km in Gossi with an extreme of more than 70 km where there is no health facility. This distance is statistically higher in Ber than in Gossi (Mann-Whitney U test *p* < 0.0001) (Fig. 2).

**Fig. 3:**
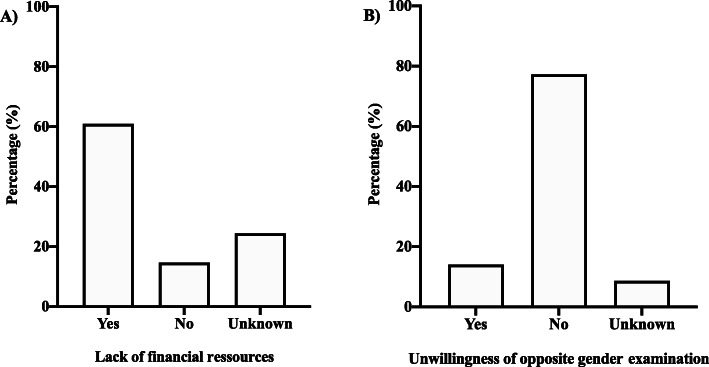
Perception of respondents about the lack of financial resources and the unwillingness of opposite gender examination

Approximately, 60% of our participants stated that they lacked the financial resources to regularly attend health services (Fig. 3a). In addition, more than 77% of respondents said they would not accept being examined by an agent of the opposite gender (Fig. 3b).

## Discussion

Most of the study population (47%) were illiterate and mainly represented by the age group of 38–47. The reluctance of some older people to enroll their children in the formal education system may be due to a loss of confidence between the country’s officials and their partners. There were more men among the study participants because the prevailing perception in these communities amongst women is that it is the men’s responsibility to represent or to speak on behalf of the community. Also, in *Tamasheq* settings, women are inaccessible to visiting men, unwilling to speak to a stranger or a man who could become her husband because of customs and being Muslim (100% of participants).

*Tamasheq* was the most widely-spoken local language (65%). *Tamasheq* is the language of the Tuareg nomads in Mali and is the common point of a whole ethnic groups known as “*Kel Tamasheq* (meaning people of the *Tamasheq* language)”. Most of them can read and write Arabic. They learn this language from an early age in order to read and write *Quran* later. *Kel Tamasheq* also have their own alphabet called “*Tifinagh* (meaning *Kel Tamasheq* alphabet). The linking with nomadic ancestral values is a key element that hinders social and health development in northern Mali. The standard of living is not very high hence; the society is based on a very strong tribal model and mutual assistance. When implementing public health interventions, the actors must take into account these factors for the success of the program [16–18]. This is likely the reason that people don’t participate in public health interventions.

In Mali, the number of qualified health personnel is inadequate throughout at all levels of the health system. It is also unequally distributed over the country (more specialized staff in the south than in the north). The same scenarios is observed for health infrastructure [19–21]. The nomadic community pays a heavy price because of the population’s lifestyle and the extent of the occupied region.

The most used means of transportation to reach the health center was a car in nearly 40% of cases. The cars used most often belong to the traders of the locality, some high-ranking officials such as the mayor, members of parliament and tribal chiefs. Unfortunately, these cars are not available for the community members all the time. In addition, it is often necessary to be a close relative of the owner to be able to borrow the car. There are also some non-governmental organizations such as *AVSF - Agronomes et Vétérinaires Sans Frontières* (https://www.avsf.org/fr/posts/570/full/mali) that operate in these areas with mobile teams to give care [11]. The cars of these organizations could also be used if the opportunity lends itself to it. Distances from referral health centers are quite long. With the sand of the Malian desert the roads are inaccessible. This demonstrates the difficulties that the populations have with the means of transport in nomadic areas. All these centers are led by nurses of both genders’ male and female except the health center of Gossi which is led by a physician. Furthermore, 41.15% of our study participants lived less than 5 km from the nearest health center. This short distance can be explained by the fact that the investigators were not able to go very far from the city of Ber which hosts a military camp. This was due to insecurity and to avoid exposure to attacks of any kind. The same situation was observed in Gossi. The cities of Gossi and Ebang imalane in the commune of Gossi, each have a health center provided by a mosque or church. Indeed, these services are available only to those who live near these centers. Very remote nomadic campsites have not been included because of the very limited means of transportation and also the insecurity.

In Ber, El Moctar et al. (2006) found that care is not available to all in this area. Indeed, those who have access to care are those who are essentially living near health centers and those with the means to ensure the costs of transportation and medicines. For others, going to the health center is difficult or even impossible because of poverty or the distance [22]. High transportation costs and the poor-quality of the roads remain one of the main barriers that limit access to health centers in this area. The long distances to health facilities and the unavailability of ambulances have exacerbated existing inequalities in access to quality care in the northern part of Mali [22].

Approximately, 39.04% of participants found that the cost of care was too expensive against 11.54% who thought that the cost of care was affordable compared to neighboring countries such as Burkina Faso or Algeria or Niger. However, there is also a cultural problem: many of these people have, cattle, and other animals but do not want to sell their animals for cash to pay for health needs. In 2006, El Moctar et al. found in Ber that 96.6% of people said they were ready to go to the health center if care was free [22]. Lack of money was cited as the main obstacle to access to health care among women respondents (53%) [12]. Many studies reveal that the most vulnerable do not receive all necessary care when the needs are greater than elsewhere. Inequality in access to care is present almost everywhere in nomadic areas even in sedentary and nomadic communities. More than 63.26% of the study participants had an average monthly income lower than 120$ USD. The cost of health care is a burden increasingly unbearable for the socially and economically vulnerable communities [11, 23, 24];.

Another factor cited was the use of traditional medicine, in which 44% of the participants visits first traditional healers and marabouts (people who use *Quran* verses to treat diseases) in case of illness. El Moctar et al. report that 57.4% of nomadic communities use traditional medicine first and modern medicine as an alternative. Although cost is important, this factor should not be overestimated in the analysis of the conditions of access to basic health services. Many studies have shown the link between improved quality and increased attendance within nomadic populations [20, 25, 26].

Advanced strategy consists of offering to villages or neighborhoods far from the health facility a health care team that provides the necessary elements such as vaccinations, prenatal care, and education sessions for newborns. It makes it possible to reach people who cannot regularly travel to the health center. The advanced strategy generally covers localities 5 to 15 km from the community health center. Mobile strategy usually refers to health staff who travel from the community health center to provide health services to people living in remote areas or to mobile populations such as nomads. Mobile teams may travel for several days to reach targeted people. In Mali, this strategy is recommended beyond 15 km. The two strategies are currently used to reach non-functional health areas and are often considered the best approach in nomadic areas. An evaluation made by AVSF reported that 84% of participants said that the actions of the joint mobile health component (human and animal health) were in line with their needs [27]. Furthermore, a lot of people would prefer a fixed health center for several reasons including cost, stability of personnel, health facilities, the short stays of the mobile team on-site, non-functionality of certain markets (weekly local markets), the lack of communication and community leadership. All these reasons make the above-mentioned approaches inappropriate for better access to health services. The data presented in this article will certainly have changed since the 2012 crisis, which is still ongoing and is exacerbate from day to day. We believe that access to care has become much worse than when we conducted this survey. We affirm this because immediately after our investigation, the military coup of 2012 occurred and the following year the whole north of Mali was invaded by terrorists. This remains the current state as we write this manuscript. All state services, including health services or health providers have left these regions.

Our study does have some limitations. First, the data are almost 10 years old, having been done in 2011. Nevertheless, we believe that these data are still valuable. Since we conducted this study, the health care services in northern Mali have not improved, primarily because of the coup of 2012, and secondly the Tuareg rebellion and the invasion of the country by terrorists. These conditions have disrupted health services. Today, health care services are almost non-existent in these districts where the data were collected. Reforms of the health sector are underway in Mali, but these reforms are not being implemented yet and certainly will not have any impact on the situation in the upcoming months. A new study is needed to assess health services and their accessibility by the nomadic populations 10 years after our study.

Second, the data may certainly contain information and selection bias. Some participants may have exaggerated the importance of the information just to get the investigators’ attention, or they may have hidden cultural information such as household decision-making power that could be a significant barrier to access health care. In order to minimize these biases, we have explained the questions to the participants in a very precise manner in *Kel Tamasheq* the language of the study population, and also in French, the official language of Mali. Additionally, almost 80% of the interviewers were natives of the northern regions and ethnic groups, making them more familiar with the people and their culture.

Third, due to insecurity, we were unable to visit some areas of the study sites where there are large nomadic camps. If we had been able to visit, the information reported here could have been more important. The investigation teams (interviewers) were made up of two people (one man and one woman). In some remote areas, interview teams were made up of only men to protect their integrity ant to avoid cultural problems with regard to women’s place in society. It is possible that some women would not have felt comfortable speaking with them during the study, so this is an additional limitation to our research.

Finally, we should have developed multivariate models since we are talking about factors that hinder access to health care. Unfortunately, the design of this study did not allow us to collect data to do the modeling. The primary goal of this study was not to identify risk factors but to explore the field to see how the community-directed interventions process could be implemented. It should also be noted that this study is part of a larger study. Future studies should include these aspects in their design.

## Conclusion

On completion of the study, it appears that despite the efforts from the Malian government and its health partners, access to basic health interventions is not meeting expectations in nomadic areas. This study shows the importance of a set of factors that should be considered to improve access to health interventions in nomadic areas. These factors include the underutilization of health services, quality of services offered, the attachment to ancestral nomadic values, poverty, gender and the current strategies recommended by health policies. However, nomads are interested in all strategies that will allow them to participate in the management of their health problems.

## Supplementary Information

**Additional file 1.** Questionnaire. List of questions that were used to collect the data.

## Data Availability

The datasets generated and analyzed during this study are presented in Tables 1, 2, and 3 and Figs. 1 and 2. Additional information is available from the authors upon reasonable request.

## Abbreviations

ADESAH: *Association pour le Développement Endogène au Sahel*
AVSF: *Agronomes et Vétérinaires Sans Frontières*
CHW: Community Health Workers
NGO: Non-Governmental Organization
PMA: Minimal Package of Activities
SPSS: Statistical Package for Social Sciences
USD: United States Dollar
