# Supplementary material for: Factors hindering health care delivery in nomadic communities: a cross-sectional study in Timbuktu, Mali
Source: BMC Public Health. 2021 Feb 28;21:421. doi: 10.1186/s12889-021-10481-w (PMC7916290; doi:10.1186/s12889-021-10481-w)
Supplement: Supplementary file 1 — Additional file 1. Questionnaire. List of questions that were used to collect the data. [file 12889_2021_10481_MOESM1_ESM.docx]

**QUESTIONNAIRE FOR DATA COLLECTION**

1. How old are you? _____years

2.Gender: Male_____ Female_____

3. What is your marital status?

Single___ Married______Separated______ Divorced______ Others______

4. What is your place in the family (responsibility)? In the fraction? In the village? _____ 99. To be specified .................................

5. How long have you lived in this area? __________

6. What is your local language? Tamasheq_____ Maure______ Arabe_____Bambara____ Peulh____ Other______

7. What is your level of education? Illiterate___ Basic_____ Secondary ____Technical College____ University_____ Other_______

8. What is your main occupation? -----------------------------

9. What is your average monthly income? < 60.000Fcfa____ 61.000 - 100000Fcfa______ 101000 – 175000Fcfa - 176000-249000Fcfa - 250000Fcfa

10. What are your information sources?

TV_____ Radio_____Phone______ interpersonal ____Autres_________

11. Do you know which diseases are endemic in your community?? / __/yes /___/ /no

12. If yes, which one(s)? ____________________________________________

13. Do your family members have access to health care system? Yes______ No_____

14. If yes, what kind of health care system? ___________________________________

15. And how? ___________________________________________________________

16. If not, why? _______________________________________________________

17. What methods do you use against diseases? Insecticide-Treated Net ____MDA (mass drug administration)____Hygiene: Body _____ _Food_____Vaccination______

18. If yes against which disease________________ Others________________________

19. How many people compose your family?

20. How many consultations have they done in the last 12 months at:___ CHC (community health center)____District health hospital____Hospital_____tradi-therapists_____Others______

**CURRENT METHODS FOR DISTRIBUTING DRUGS**

21.Do you know how Mali's health system is organized? Yes_____,Non_____

22. If yes, explain____National level___________________________

District level________________________________________________

CHC Level______________________________________________________

23. When was the last time you were there (hospital)

24. What is your assessment? Good____ Bad_____ Other______

25. What are the different ways to access to the health centre? ___________________________________________________________________________

26. In yout opinion, what is the best suited to a nomadic environment? _____________________

27. Have you ever been treated for a disease? Yes____ No_____

28. If yes, what type of treatment? Traditional _____ Medicine

29. If medical, have you participated to the mass drug administration? Yes_____ No____

30. If yes, against which diseases? ____1. Filariasis, 2. Schistosomiases, 3. Trachoma, 99. Others to be specified ___________________________

**THE ACCESSIBILITY OF HEALTH CENTRES FOR THE LOCAL POPULATION:**

31. Is there a health centre in your community? Yes____ No_____

32. If yes, what is the status? Good____ Bad____ Acceptable____

33. What do you think of the staff? Good____ Bad____ Sufficient____ Insufficient____

34. What do you think of the drugs? Good____ Bad___Sufficient____ Insufficient___

35. Do women and children have access to the health centre as well as men? Yes____ No_____

36. If not why not?_____________________________________________________

37. How many miles separate you from the nearest health centre? Less than 5 km ____between 5-10 km____ 10-20 km____ more than 20 km_____

38. What are your patients transporting methods? Car____ camel_____ foot_______ Carriage _______Others____________________________________

39. Do you consult traditional healers? Yes____ No______

40. If yes, why?_____________________________________________

41. On average how many times a year for you____ your wife_____ your children_____

42. What protects you against illness? God____ Health worke______________ Fetish_________Others___________________________

43. What types of problems do you encounter in health centres? ___________________________________________________________________________

44. For what types of illnesses do you go to health centres? ___________________________________________________________________________

45. For what reasons do you not go to the health centre: distance /____/ cost /____ / quality of care /____ / waiting time /_____/lack of ressources /____/ Others (to be specified)

46. Which sections of the population have the least access to the health centre in your opinion? Men____ Women______ Older people_______ Younger adults____ Children_____ Other_______ Why? ____________________________________________

47. What proposal do you make to the Malian government and partners to facilitate access to community-based health interventions for nomadic and rural communities? ____________________________________________________________________________________________________________________________________________________________________

**EVALUATE STRATEGIES AND PROCESSES FOR ACCESSING community based interventions**

48. Is care (consultation, medication) available at any time? Yes____No_______

49. If yes, what is the cost? Too expensive______ a little expensive______ affordable____ cheaper______ Others (to be specified)..............................………………………………………………

50. Is the provision of care accompanied by advice (hygiene, dosage and against the indication of medicines)? Yes____ No____

51. Is there a follow-up? (observance, monitoring, appointments...) Yes____ No____

1. What are the drug administration ways that are most accepted by people? Intra-Veinus injection____Intra Muscular injection____ Oral____ Topique____ Other____
2. Give the reasons for your choice__________________________________
3. Do you want health workers? Male____ Female_____ Mixed_______
4. Will you be willing to be consulted by the opposite gender? Yes____No____
5. Why?...........................................................................................................................
6. Do you prefer a health worker from your community? Yes____No___
7. Why? ________________________________________________________
8. Have you ever used self-medication with modern medicines? Yes____No____
9. If yes, how many times in the previous 3 months? ___________
10. What types of drugs do you use most often?________________________
11. Against what types of affections? Cite:___________________________________
12. What do you think is the best strategy for providing care in a nomadic environment? Fixed centre____ Advanced strategy ____Mobile team ____ Mixed mobile team (Man and Animale)________ Others____________
13. Are there any local organizations in your community that are interested in health issues/_____/ yes/____ / no
14. If yes How much /___/
15. whats types?........................................................................... ...
16. Since when?..............................................................................................................
17. Have you adhered to it: No /____/ Little /_____ / Completely/_____ /

**We are at the end of our discussion, please let me know if you have any questions or comments to make?**

**End of the interview, thank you** !
